# Einführung des Entlassmanagements an einer Universitätsklinik für Chirurgie: Explorative Analyse von Kosten, Verweildauer und Patientenzufriedenheit

**DOI:** 10.1007/s00103-022-03497-z

**Published:** 2022-02-09

**Authors:** Volker Aßfalg, Sophia Hassiotis, Marion Radonjic, Sarah Göcmez, Helmut Friess, Elke Frank, Jörg Königstorfer

**Affiliations:** 1 Klinik und Poliklinik für Chirurgie, Klinikum rechts der Isar, TU München, Ismaningerstr. 22, 81675 München, Deutschland; 2 Lehrstuhl für Sport- und Gesundheitsmanagement, TU München, München, Deutschland; 3 Finanzcontrolling, Klinikum rechts der Isar, TU München, München, Deutschland; 4 Kaufmännische Direktion, Zentrale Steuerung Entlassmanagement, Klinikum rechts der Isar, TU München, München, Deutschland; 5 Kaufmännische Direktion, Klinikum rechts der Isar, TU München, München, Deutschland

**Keywords:** Entlassung, Stationär, Überleitung, Effektivität, Drehtüreffekt, Patient care, Inpatient, Effectiveness, Transition, Revolving door effect

## Abstract

**Hintergrund:**

Seit Oktober 2017 ist ein strukturiertes Entlassmanagement zur Überleitung von Patienten aus dem stationären in den ambulanten Sektor gem. § 39 Abs. 1a S. 10 SGB V gesetzlich vorgeschrieben. Umsetzung und Finanzierung obliegen dem behandelnden Krankenhaus.

**Ziel der Arbeit:**

Ermittlung der Kosten des Entlassmanagements, Feststellung der Patientenzufriedenheit mit der Überleitung, Messung der Wiederaufnahmerate (Drehtüreffekt) sowie Vergleich der mittleren Krankenhausverweildauer vor und nach Einführung des Entlassmanagements in der Chirurgischen Klinik der Technischen Universität München.

**Methoden:**

Einjahreskostenanalyse, retrospektive Analyse der Krankenhausverweildauer vor und nach Einführung des Entlassmanagements, Patienteninterviews zur Untersuchung der Zufriedenheit und Beurteilung der Qualität des Entlassmanagements.

**Ergebnisse:**

Die Kostenanalyse ergab pauschale Kosten in Höhe von 43 € pro Patienten bzw. 391 € bei komplexem poststationären Versorgungsbedarf. Eine statistisch signifikante Verkürzung der Verweildauer durch das Entlassmanagement ließ sich in 3 Subgruppen nicht nachweisen. Die Qualität der Überleitung wurde mit Schulnoten von durchschnittlich 1,8 bis 1,9 benotet. Der Drehtüreffekt wurde bei 3,4 % der Patienten festgestellt.

**Diskussion:**

Das analysierte Entlassmanagement stellt eine effektive und qualitativ erfolgreiche, jedoch kostenverursachende Maßnahme dar, die mittelfristig im Rahmen der Fallpauschalenvergütung im G‑DRG-System (German Diagnosis Related Groups) Berücksichtigung finden und voraussichtlich kostensteigernd wirken wird. Eine an die Überleitungsqualitäten der verschiedenen medizinischen Fachbereiche angepasste, fallspezifische Vergütung des Entlassmanagements könnte einen Lösungsansatz darstellen, um die Bedürfnisse verschiedener Anspruchsgruppen zu berücksichtigen.

**Zusatzmaterial online:**

Zusätzliche Informationen sind in der Online-Version dieses Artikels (10.1007/s00103-022-03497-z) enthalten.

## Abstract (en)

**Background:**

Discharge management has been mandatory by law in Germany since October 2017, and hospitals are required to finance and implement this. Currently there are no data available on the costs and effects of discharge management on the length of hospital stay.

**Aims:**

Determination of the costs of discharge management in the Department of Surgery at the University Hospital rechts der Isar of the Technical University of Munich, Germany, assessment of the length of stay in comparison with and without discharge management, and evaluation of patients’ satisfaction to create first precedents for future negotiations about adequate financing.

**Methods:**

Cost analysis of discharge management in the Department of Surgery at the School of Medicine at the Technical University of Munich, retrospective analysis of the mean length of hospital stays before and after implementation of discharge management, and patient surveys on the quality of the structured transition process and their satisfaction.

**Results:**

The cost analysis revealed lump costs of € 43 per patient and € 391 for patients with a need for complex management. No statistically significant shorter length of hospital stay after the implementation of discharge management was found by analyzing three patient subgroups. The overall rate of patients returning to the hospital due to complications associated with the surgical procedure was 3.4%.

**Discussion:**

Discharge management in the Department of Surgery at the hospital is an effective and potentially quality-enhancing but at the same time cost-driving measure, which, in the medium term, will enter G‑DRG rates and may thus increase costs. A possible solution to meet various stakeholders’ needs could be a case-specific financial remuneration of discharge management that is adapted to the transition qualities of the various medical departments.

## Einleitung

Der zum 01.10.2017 in Kraft getretene, durch die Deutsche Krankenhausgesellschaft (DKG), die Kassenärztliche Bundesvereinigung (KBV) sowie den Spitzenverband der gesetzlichen Krankenversicherungen (GKV-Spitzenverband) vereinbarte Rahmenvertrag über ein Entlassmanagement und seine mitgeltenden Vereinbarungen stellten die Krankenhäuser vor strukturelle, personelle und finanzielle Herausforderungen. Aktuell liegt der Rahmenvertrag gemäß § 39 Abs. 1a S. 10 Fünftes Buch Sozialgesetzbuch (SGB V) in der 5. Fassung vor.

Das Ziel nach § 2 des Rahmenvertrages, jeden Patienten individuell, bedürfnis- und ressourcenorientiert lückenlos vom Krankenhaus in die ambulante Weiterversorgung überzuleiten [1], ist aus Sicht von Patienten und Public Health wünschenswert. Aber auch sozioökonomisch ist eine gelungene Überleitung in den weiterbehandelnden Sektor erstrebenswert. Ziel ist es, durch eine medizinisch-pflegerisch gut abgestimmte und zum richtigen Zeitpunkt durchgeführte Überleitung in die vorbereitete Nachversorgung die Krankenhausverweildauer zu minimieren und eine Wiederaufnahme in das Krankenhaus aufgrund einer Verschlechterung oder einer Komplikation (sog. Drehtüreffekt) zu vermeiden. Eine niedrige Rate an wiederkehrenden Patienten gilt als Maß für eine sichere Überleitung in den ambulanten Sektor.

Der patientenseitige Anspruch auf die Dienstleistung zur Organisation der Überleitung besteht ausschließlich gegenüber dem entlassenden Krankenhaus [1]. Die Vorgaben eines strukturierten Entlassmanagements sind im Rahmenvertrag niedergelegt. Die individuelle Umsetzung kann von jedem Krankenhaus oder auch jeder Abteilung innerhalb eines Krankenhauses fachspezifisch und bedarfsorientiert erfolgen. Dies lässt eine hohe Heterogenität der Strukturen, Arbeitsintensität und Kosten der einzelnen Entlassmanagementeinrichtungen zwischen den Krankenhäusern, aber auch innerhalb eines Krankenhauses zwischen verschiedenen Abteilungen erwarten.

Die Vergütung des Entlassmanagements im Rahmen der Fallpauschalen des G‑DRG-Systems (*German Diagnosis-related Groups System*: an Diagnosen geknüpftes Fallpauschalensystem) wird in Zukunft gemäß der InEK(Institut für das Entgeltsystem im Krankenhaus)-Kostenträgerrechnung erfolgen. Bisher existieren noch keine Publikationen zu den tatsächlichen Kosten in den Krankenhäusern.

In der vorliegenden Studie wurde das Entlassmanagement an der Klinik für Chirurgie des Universitätsklinikums rechts der Isar der TU München in einer für diese Klinik charakteristischen Kohorte untersucht. Ermittelt wurde, welche Kosten durch das multimodale Entlassmanagement verursacht werden, wie hoch diese sind und ob sich im Vergleich zu der Zeit vor der Einführung des Entlassmanagements eine Verkürzung der Verweildauer ergeben hat, z. B. durch einen Abschluss der Wundtherapie oder eine Stomaeingewöhnung im ambulanten Bereich. Außerdem erfolgte mittels einer Patientenbefragung die Evaluation, wie effektiv und qualitativ zufriedenstellend die Strukturen des Entlassmanagements arbeiten.

## Methoden

Die Klinik und Poliklinik für Chirurgie des Klinikums versorgt jährlich durchschnittlich 3500 stationäre Patienten (Mittelwert 2016–2020) mit dem gesamten Spektrum der Allgemein- und Viszeralchirurgie. Die Schwerpunkte liegen auf der onkologischen Chirurgie von Pankreas und hepatobiliärem System, des oberen Gastrointestinaltrakts sowie der Kolorektal- und Sarkomchirurgie. Seit 01.02.2017 erfüllt die Klinik für Chirurgie die Anforderungen an das standardisierte Entlassmanagement nach § 39 Abs. 1a S. 10 SGB V gemäß dem Rahmenvertrag in vollem Umfang. Das Entlassmanagement wurde zwischen dem 01.05.2016 und dem 31.01.2017 stufenweise aufgebaut und in die Klinikabläufe implementiert. 2 Mitarbeiter mit der Ausbildung in Gesundheits- und Krankenpflege mit einer Wochenarbeitszeit von zusammen 67,38 h sowie ein Student mit einer wöchentlichen Arbeitszeit von 20 h koordinieren die Prozesse des Entlassmanagements. Dies entspricht einer Wochenarbeitszeit von 87,38 h oder 2,27 Vollkräften (VK) gemäß TV‑L (Tarifvertrag für den öffentlichen Dienst der Länder).

Der zu erwartende Bedarf einer poststationären Versorgung (poststationäres Versorgungsproblem [pVP]) eines jeden Patienten wird mittels eines Ampelsystems im Initialscreening zu Beginn des Aufenthaltes von den Mitarbeitern des Entlassmanagements standardisiert erfasst.

**Figure 1:**
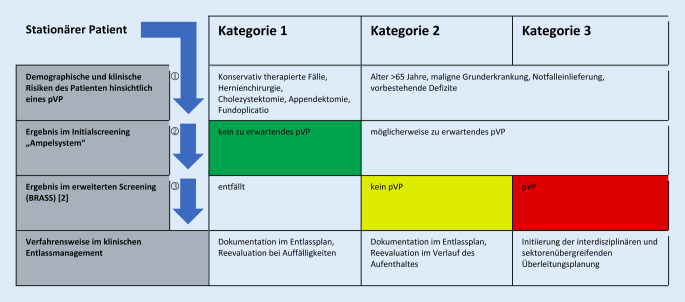

Bei Patienten, die im Initialscreening ein möglicherweise zu erwartendes pVP aufweisen, wird ein differenziertes Assessment analog dem Blaylock Risk Assessment Screening Score (BRASS; [2]) durchgeführt und es werden ggf. nötige Maßnahmen zur Überleitung in den ambulanten Bereich oder die stationäre Weiterbehandlung eingeleitet (Abb. 1 und 2).

**Figure 2:**
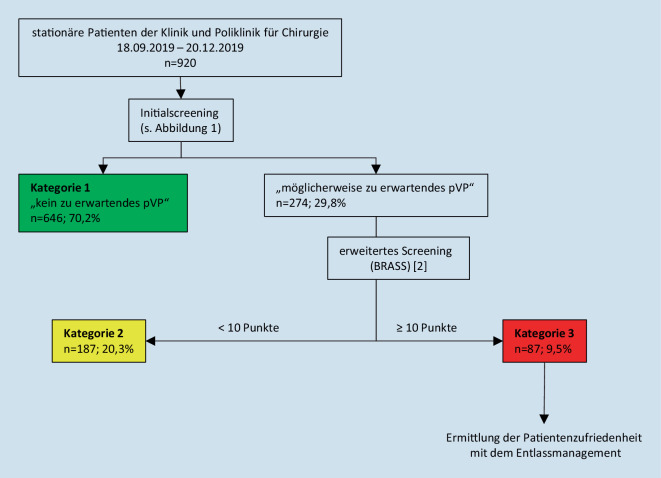

### Ermittlung der Kosten des Entlassmanagements

Alle Patienten, die innerhalb eines dreimonatigen Zeitraums (18.09.2019–20.12.2019) in der Klinik und Poliklinik für Chirurgie eine stationäre medizinische Behandlung erfahren haben (*n* = 920), wurden hinsichtlich der Kosten, die im Rahmen des Entlassmanagements anfielen, betrachtet. Für die Kostenermittlung wurden die 3 großen Kostenblöcke (Personalkosten, Kosten im Wirtschafts‑/Verwaltungsbedarf, Gemeinkosten/Infrastrukturkosten) analysiert. Die Vorgehensweise der Kostenermittlung entspricht hierbei der InEK-Kostenträgerrechnung:

- Die Personalkosten ergaben sich aus einer Arbeitszeitpersonalbedarfsplanung unter Berücksichtigung der für die jeweiligen Berufsgruppen tariflichen Bedingungen. Der durchschnittliche Zeitaufwand der einzelnen jeweils an der Versorgung beteiligten Personengruppen für einen Patienten pro Aufenthalt im Rahmen des Entlassmanagements erfolgte mittels Zeitmessung bei 30 (34 %) randomisierten Patienten der Kategorie 3 (Abb. 2). Die Verteilung der Diagnosen dieser Stichprobe entsprach der Verteilung dieser Diagnosen im Patientengut der Klinik. Aus den Arbeitszeitstunden pro Jahr, welche mithilfe der Personaldurchschnittskosten für die einzelnen Berufsgruppen und der Messung der Behandlungszeit je Patienten ermittelt wurden, ergaben sich die Personalkosten je Patienten.
- Die Kosten im Wirtschafts‑/Verwaltungsbedarf wurden mit einer Pauschale i. H. v. 2,00 € pro Patienten angesetzt. Dieser Betrag beinhaltet alle Kosten, die für den Dokumentaufwand anfallen. Es handelt sich hier um einen Durchschnittspreis.
- Die Gemeinkosten/Infrastrukturkosten enthalten sämtliche Kosten des nichtmedizinischen Bereichs (ziviler Bereich), wie z. B. Energie/Strom, Wasser, Reparaturarbeiten und Informationstechnologie (IT). Daraus ergibt sich ein monatlicher durchschnittlicher m^2^-Preis, welcher mit der Arbeitsfläche (Büroraum des Entlassmanagements) multipliziert wurde.

**Figure 3:**
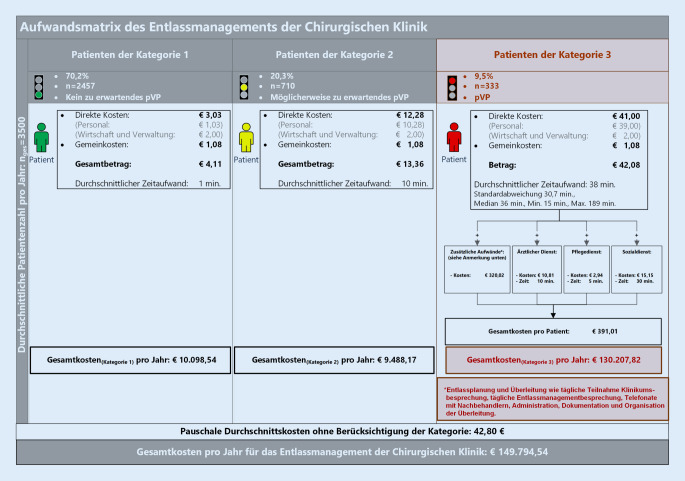

Die Kosten pro Patienten wurden anschließend auf ein Jahr extrapoliert (Abb. 3).

### Ermittlung der Patientenzufriedenheit mit dem Entlassmanagement

Alle 87 Patienten der Kategorie 3 willigten in die Teilnahme an der Analyse ein und wurden im Rahmen eines standardisierten Telefoninterviews 10–14 Tage nach der Entlassung aus der stationären Behandlung befragt. Der Interviewfragebogen zur Analyse der Patientenzufriedenheit mit dem Entlassmanagement ist im Onlinematerial zu diesem Beitrag zu finden.

**Figure 4:**
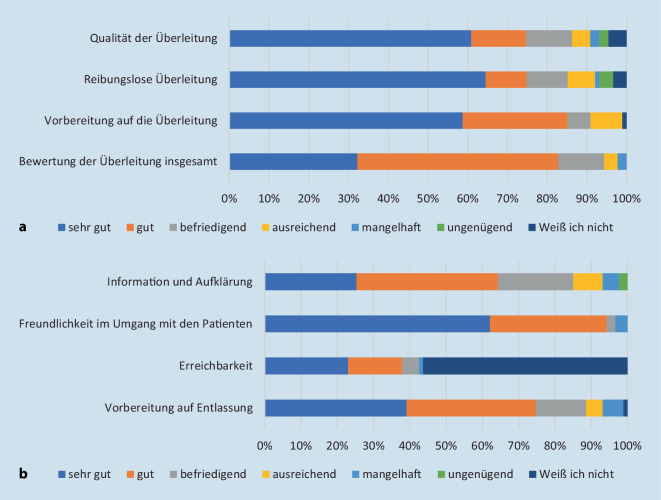

Mittels 4 Fragen wurde auf einer sechsstufigen Skala (1 = beste Bewertung, 6 = schlechteste Bewertung; Reliabilität: Cronbachs Alpha = 0,77) die Zufriedenheit mit dem Entlassmanagement bezüglich der Vorbereitung auf die Zusammenarbeit der Leistungserbringer erfasst (Abb. 4a). In gleicher Art und Weise wurden die Freundlichkeit, Erreichbarkeit und Informationsqualität des Entlassmanagements mittels 4 Fragen ermittelt (Cronbachs Alpha = 0,76; Abb. 4b).

### Ermittlung des Drehtüreffektes

Die befragten Personen wurden weiterhin gebeten, anzugeben, ob sie in den ersten 10 Tagen nach der Entlassung aus dem Klinikum bei einem Hausarzt, Facharzt oder in einer Krankenhausambulanz waren. Die häufigsten chirurgischen Komplikationen, die mit dem Eingriff in Zusammenhang stehen und zu einer Wiederaufnahme führen könnten (Wundinfektionen, Komplikationen der Anastomose, Darmparalyse/-passageprobleme), treten typischerweise mit einer hohen Wahrscheinlichkeit in diesem Zeitraum auf [3]. Zusätzlich wurden die Patienten gefragt, ob sie ungeplant stationär in einem Krankenhaus aufgenommen wurden. Falls sie dies bejahten, wurden sie gefragt, ob der ungeplante stationäre Aufenthalt in Zusammenhang mit der vorangegangenen Behandlung stand (Kriterium des Drehtüreffektes) oder auf eine andere, neue Diagnose zurückzuführen war.

### Ermittlung des Effekts des Entlassmanagements auf die Krankenhausverweildauer

Ein Pre-Post-Design wurde angewendet. Alle Patienten, die im Zeitraum 01.01.2014–30.04.2016 vor (= Pre-Gruppe) bzw. zwischen 01.02.2017 und 31.10.2019 nach der Einführung des Entlassmanagements (= Postgruppe) in der Klinik eine stationäre medizinische Behandlung erfahren hatten, wurden retrospektiv erfasst.^1^

Eingeschlossen wurden die Patienten mit der Hauptdiagnose ICD-10-GM 2019 C20 (Rektumkarzinom) mit den G‑DRG-Operationen- und Prozedurenschlüsseln OPS 2019 5‑484 oder 5‑485 zusammen mit 5‑462 (tiefe anteriore Rektumresektion und Ileostomaanlage bzw. abdominoperineale Rektumamputation und Descendostomaanlage). Die zweite Analyse umfasste die Patienten mit der Hauptdiagnose C16 (Magenkarzinom) und dem OPS 5‑473 (totale Gastrektomie). Außerdem wurden die Patienten mit der Nebendiagnose T81.3 und/oder T81.4 (postoperative Wundinfektion) untersucht. Diese 3 Behandlungsdiagnosen wurden ausgewählt, da für sie in der Regel eine Nachversorgung organisiert werden muss:

- Rektumkarzinom: Stomaüberleitung,
- Magenkarzinom: Ernährungstherapie,
- Wundheilungsstörung: Wundversorgung.

Die Verweildauer der untersuchten Patienten wurde jeweils dem Krankenhausinformationssystem entnommen. Als Prädiktoren der Verweildauer wurden neben der Einführung des Entlassmanagements das Alter der Patienten und die effektive Bewertungsrelation (EffBewR) aufgenommen.

Die statistischen Berechnungen erfolgten mittels der Software IBM® SPSS® Statistics 28.0 (IBM, 1 New Orchard Road, Armonk, New York 10504-1722, United States). Für die Vergleiche zwischen Pre- und Postgruppe wurden für die 3 Kohorten separate ANOVAs (ANalysis Of VAriance; Kontrollvariablen: Alter und EffBewR) durchgeführt. Ein *p*-Wert < 0,05 wurde als statistisch signifikant gewertet.

## Ergebnisse

### Analyse des prospektiv untersuchten Patientenkollektivs

Im Beobachtungszeitraum vom 18.09.2019 bis 20.12.2019 wurde bei 646 Patienten (70,2 %) kein Bedarf einer Überleitung nach Entlassung (Kategorie 1) festgestellt und 274 Patienten (29,8 %) wurden dem standardisierten BRASS [2] unterzogen. Bei einem Wert von ≥ 10 Punkten oder der sekundären Notwendigkeit einer poststationären Versorgung im Verlauf des stationären Aufenthalts wurde eine Betreuung durch das Entlassmanagement veranlasst (Kategorie 3). Erst ab dieser Etappe wurden auch der ärztliche Dienst, der Pflegedienst auf der Station und der Sozialdienst sowie sekundär die Nachversorger des ambulanten Sektors bezüglich einer Überleitung aktiv (Abb. 1). Dies war bei 87 Patienten (9,5 %) indiziert (Abb. 2).

Bei den durchgeführten Zeitmessungen der randomisiert ausgewählten Patienten benötigten die Mitarbeiter des Entlassmanagements durchschnittlich eine Minute für das Initialassessment und 10 min für den Fall, dass zusätzlich ein erweitertes Assessment (BRASS) erforderlich wurde (Abb. 2). Für Patienten mit einem pVP (Kategorie 3) lag der Zeitaufwand des Personals im Entlassmanagement allein für Patienten- und Angehörigenkontakte bei durchschnittlich 38 min (Mittelwert 38,0 min; SD 30,7; Median 36 min; Min. 15; Max. 189 min). In dieser Gruppe von Patienten waren nun zusätzliche Aufwände zur Entlassplanung und Überleitung, wie die tägliche Teilnahme an der Klinikbesprechung, tägliche Entlassmanagementbesprechungen, Telefonate mit Nachbehandlern, Organisation und Koordination der Überleitung sowie Administration und Dokumentation, erforderlich, sodass sich eine Vollauslastung des Personals des chirurgischen Entlassmanagements mit 2,27 VK gemäß TV‑L ergab. Für die beiden letztgenannten Tätigkeiten wurde eine studentische Hilfskraft eingesetzt (Abb. 3). Die Patienten der Kategorie 3 wiesen eine mittlere effektive Bewertungsrelation (EffBewR) von 4,04 (Median: 3,42 [0,21-15,37]) auf, wohingegen diese bei den Patienten der Kategorien 1 und 2 bei 2,84 (Median: 1,25 [0,21-64,0]) lag.

### Kosten des Entlassmanagements

Die Personal‑, Wirtschafts‑, Verwaltungs- und Infrastrukturkosten sowie die Berechnung der Pauschalkosten im Entlassmanagement pro Fall ohne Berücksichtigung des individuellen zeitlichen Aufwandes ergaben im Durchschnitt rund 43 €.

Die Differenzierung der Fälle zeigte, dass sich die durchschnittlichen Kosten für Patienten der Kategorie 1 auf ca. 4 € beliefen. Für einen Patienten der Kategorie 2 entstanden Kosten in Höhe von ca. 13 € und in der Kategorie 3 entstanden Kosten von ca. 391 € pro Patienten (Abb. 3).

### Patientenzufriedenheit mit dem Entlassmanagement

Die Vorbereitung auf die Überleitung in den ambulanten Sektor wurde auf einer Skala von 1–6 analog der Schulnotengebung mit einem Mittelwert von 1,8 (Abb. 4a) bewertet. Die Informationsqualität, Freundlichkeit und Erreichbarkeit des Entlassmanagements wurden insgesamt mit einem Mittelwert von 1,9 benotet (Abb. 4b). Vergleichbare Daten zur Patientenzufriedenheit vor Einführung des strukturierten Entlassmanagements lagen nicht vor.

### Drehtüreffekt

Bei 4 Patienten (4,6 %) war ein ungeplanter erneuter stationärer Aufenthalt innerhalb von 10 Tagen nach Entlassung erforderlich. Die Patienten kamen hierzu wieder in das Klinikum zurück und bei 3 dieser Patienten (3,4 %) stand der Aufnahmegrund im Zusammenhang mit dem ersten Aufenthalt. Vergleichbare Daten zu stationären Wiederaufnahmen vor Einführung des strukturierten Entlassmanagements lagen nicht vor.

### Effekt des Entlassmanagements auf die Krankenhausverweildauer

In Tab. 1 sind die Ergebnisse der Verweildaueranalyse der 3 Kohorten, einschließlich der Daten für das Alter der Patienten, und die EffBewR dargestellt. Bei den Patienten nach Resektion eines Rektumkarzinoms zeigte sich eine marginal nicht signifikant (*p* = 0,07) kürzere Verweildauer in der Gruppe nach Einführung des Entlassmanagements (unter Einbezug der Kovariaten Alter und EffBewR, welche beide einen positiven Zusammenhang mit der Verweildauer aufweisen). Dahingegen konnte nach Gastrektomie bei Magenkarzinom bzw. bei Nachweis einer Wundheilungsstörung jeweils keine Änderung der Verweildauer nach (vs. vor) Einführung des Entlassmanagements nachgewiesen werden.

**Table 1:** 

	Fallzahl	VWD MW	Median	Alter MW	Median	EffBewR Summe	MW	Median
*Rektumkarzinom*								
Ohne Entlassmanagement	30	20,87	13,00	58,00	59,00	153,27	5,11	3,59
Mit Entlassmanagement	40	18,30	15,00	62,28	63,50	156,77	3,92	3,60
ANOVA-Ergebnisse F (3, 66) = 103,51, *p* < 0,001, R^2^ = 0,83	Effekt der Einführung des Entlassmanagements: F (1, 66) = 3,32, *p* = 0,07			F (1, 66) = 4,36, *p* = 0,04		F (1, 66) = 320,13, *p* < 0,001		
*Magenkarzinom*								
Ohne Entlassmanagement	46	17,89	14,50	57,73	60,50	228,77	4,97	4,48
Mit Entlassmanagement	45	27,33	15,00	63,42	65,00	356,09	7,91	4,54
ANOVA-Ergebnisse F (3, 87) = 103,51, *p* < 0,001, R^2^ = 0,78	Effekt der Einführung des Entlassmanagements: F (1, 87) = 0,14, *p* = 0,71			F (1, 87) = 3,12, *p* = 0,08		F (1, 87) = 280,92, *p* < 0,001		
*Wundheilungsstörung*								
Ohne Entlassmanagement	219	39,82	29,00	62,04	63,00	1995,66	9,11	4,57
Mit Entlassmanagement	268	43,31	32,00	64,16	66,00	2880,33	10,75	5,14
ANOVA-Ergebnisse F (3, 483) = 521,01, *p* < 0,001, R^2^ = 0,76	Effekt der Einführung des Entlassmanagements: F (1, 483) = 0,08, *p* = 0,78			F (1, 843) = 0,38, *p* = 0,54		F (1, 483) = 1552,78, *p* < 0,001		

*MW* Mittelwert, *VWD* Verweildauer, *EffBewR* effektive Bewertungsrelation

## Diskussion

Der „Expertenstandard Entlassungsmanagement in der Pflege“ des Deutschen Netzwerks für Qualitätsentwicklung in der Pflege (DNQP) gibt gemäß § 113a Elftes Buch Sozialgesetzbuch (SGB XI) Empfehlungen zu Maßnahmen zur systematischen Vermeidung von Versorgungsbrüchen bei der Patientenentlassung durch eine gezielte Vorbereitung von Patienten und Angehörigen sowie einen interprofessionellen, verbesserten Informationsaustausch [4]. Andere Gesundheitssysteme, wie z. B. das US-amerikanische, kanadische oder australische, basieren seit Jahren auf einem starken ambulanten Sektor mit Überleitung aus dem Krankenhaus durch ein „discharge planning“ (dt.: Entlassungsplanung). Sie sind jedoch insgesamt grundlegend anders aufgebaut, sodass nach möglichst früher stationärer Entlassung eine kontrollierte Versorgung im häuslichen Bereich über spezialisierte Pflegekräfte erfolgt („nurse home visits“). Einige Studien aus diesen Ländern, die aus verschiedenen, meist spezialisierten medizinischen Fachbereichen stammen, konnten neben der Identifikation von Risikofaktoren für eine stationäre Wiederaufnahme kurz nach Entlassung [3, 5, 6] positive Effekte bezüglich Patientenzufriedenheit und Reduktion von Morbidität, Mortalität und Drehtüreffekt, aber auch kontroverse Auswirkungen auf die Kosten für das Gesundheitssystem nachweisen [5, 7–15]. Ein umfassendes Cochrane-Review fand keine eindeutig überzeugenden Vorteile der Einführung eines Entlassmanagements in Hinblick auf die erhofften Effekte kürzere Verweildauer, Reduktion des Drehtüreffektes und sozioökonomische Kostenreduktion. Insgesamt waren die darin untersuchten Patientenkollektive und Krankheitsbilder jedoch sehr heterogen und die definierten Studienendpunkte in sämtlichen Kategorien sehr unterschiedlich. Lediglich eine höhere Patientenzufriedenheit durch eine standardisierte Entlassungsüberleitung war überwiegend konstatierbar [16].

In Deutschland liegen bisher keine wissenschaftlichen Publikationen zur Untersuchung von Entlassmanagementstrukturen hinsichtlich Kosten, Verweildaueranalyse und Drehtüreffekt vor. Die vorliegende Arbeit zielte darauf ab, diese Forschungslücke zu schließen. Dies ist notwendig, da die Gesundheitssysteme anderer Länder sich in wesentlichen Aspekten, insbesondere auch bezüglich des Zusammenspiels von stationärem und ambulantem Sektor, vom deutschen System unterscheiden, sodass hier keine validen Schlussfolgerungen übertragen werden können.

Umfang und Kosten für die Erfassung, Betreuung, Versorgung und allgemeine sowie teilweise hochspezifische Überleitung in den ambulanten Sektor hängen von zahlreichen Faktoren ab. Beispielsweise gestaltet sich das Spektrum einer Überleitung aus der stationären Versorgung der inneren Medizin, Geriatrie oder Neurologie mit Ernährungstherapie, Dekubitusprophylaxe, Inkontinenzversorgung und Heimsauerstoffversorgung anders als bei einem chirurgischen Fach, in dem insbesondere die postoperative Wundversorgung zumindest kurz- oder mittelfristig im Vordergrund steht. Die hier vorliegende erstmals publizierte systematische Kostenanalyse für ein Entlassmanagement basiert somit auf einer sehr speziellen universitären Subgruppe aus der Allgemein- und Viszeralchirurgie mit einem hohen Anteil komplexer Fälle und ist nicht ohne Weiteres auf andere Disziplinen oder Kliniken anderer Versorgungsstufen übertragbar. Dies ist eine Limitation der vorliegenden Studie.

Die pauschalen Durchschnittskosten von ca. 43 € sowie die Kosten von rund 391 € für Patienten mit pVP (Kategorie 3) in dem betrachteten Patientenkollektiv (Abb. 3) demonstrieren den Kostenumfang, den die Klinik trägt. Die Bindung von Personal für das im Rahmenvertrag festgelegte, umfassende und strukturierte Entlassmanagement wird erst künftig durch die Neukalkulationen der DRGs zeitversetzt refinanziert werden. Im Rahmen der InEK-Kostenkalkulation ergibt sich hier systembedingt eine Latenz von 2 Jahren. Es sei erwähnt, dass in vielen Kliniken bereits vor der gesetzlichen Verpflichtung zur Durchführung eines Entlassmanagements Strukturen zur Überleitung bestanden, von denen anzunehmen ist, dass diese in die Fallpauschalenberechnungen einbezogen wurden. In zukünftigen Studien könnte beispielsweise untersucht werden, inwieweit Zusatzkosten durch zusätzliche Bürokratie entstanden sind und inwieweit diese zu Qualitätszunahmen führten [17, 18].

Neben einer Entlassmanagementpauschale für jeden stationär behandelten Patienten („Einheitspauschale“) wäre z. B. eine gestaffelte Vergütung entsprechend der Kategorien 1–3 in unserer Untersuchung denkbar. Diese Vorgehensweise könnte dem tatsächlichen Aufwand des Entlassmanagements und den jeweils damit verbundenen Kosten bereits gerechter werden.

Darüber hinaus ist auch eine aufwandsgerechte Vergütung für das Entlassmanagement in den Krankenhäusern innerhalb des Fallpauschalensystems möglich, die über die InEK-Kostenmatrix unterschiedliche Sätze für die einzelnen G‑DRGs berücksichtigt. Dabei würden Parameter wie die entlassende Fachdisziplin, die EffBewR und triggernde Faktoren, wie z. B. Komorbiditäten, widergespiegelt, um der Kostendeckung gerecht zu werden. Die Berücksichtigung der Entlassmanagementkosten in künftigen Fallpauschalen könnte – eine umfassende und an die verschiedenen Kliniken und Fachbereiche angepasste Kostenkalkulation vorausgesetzt – sogar noch präziser sein als die auf den in dieser Arbeit untersuchten 3 Kategorien basierende, gestaffelte Vergütung.

Die Gegenüberstellung der korrespondierenden, erzielten DRG-Erlöse mit den für diese Kohorte ermittelten durchschnittlichen Entlassmanagementkosten i. H. v. 391 € ergab rechnerisch im Mittelwert einen Kostenanteil des Entlassmanagements von 4,8 % (Median: 3,2 %, Min. 0,7 %, Max. 53,5 %). Dies zeigt die inhomogene Spannbreite der Kosten und unterstreicht die Notwendigkeit einer subtilen InEK-Kostenmatrix zur möglichst exakten Erfassung aller Aufwendungen.

Unsere Studie zeigte, dass trotz eines Entlassmanagements keine Verweildauerverkürzung in den hier untersuchten Fällen nachweisbar war (mit Ausnahme eines marginal nicht signifikanten Effekts in der Gruppe nach Resektion eines Rektumkarzinoms). Als Limitation ist anzumerken, dass die Patientengruppe, bei der die Resektion eines Rektumkarzinoms erfolgte, die kleinste innerhalb der 3 Krankheitsbilder war und dass weitere Kontrollvariablen, wie Geschlecht, vorangegangene Krankenhausaufenthalte und Vorerkrankungen, nicht berücksichtigt werden konnten. Künftige Studien könnten für solche Variablen zusätzlich kontrollieren. Die durchgeführte Kostenberechnung an einer verhältnismäßig kleinen Analysegruppe identifiziert das Entlassmanagement als eine kostenintensive Dienstleistung. Dieser Faktor kann als Grund für eine nur zögerliche oder suboptimale Umsetzung des Entlassmanagements angesehen werden [19]. In der vorliegenden Studie wäre eine Ermittlung der Überleitungskosten vor Einführung des strukturierten Entlassmanagements zur Feststellung der zusätzlich entstandenen Kosten wünschenswert gewesen; retrospektiv können diese nicht ermittelt werden. Dies ist eine Limitation der Studie, die in künftigen Forschungsarbeiten und an größeren Patientenkollektiven behoben werden könnte.

Der Drehtüreffekt erscheint mit 3,4 % der entlassenen Patienten im Rahmen des universitären chirurgischen Patientenquerschnitts der Klinik als zufriedenstellend und international vergleichbar [3, 20] und belegt die Überleitungsqualität durch das Entlassmanagement in den ambulanten Sektor. Die Patientenzufriedenheit im Telefoninterview bestätigt dies, auch wenn Verzerrungen durch sozial erwünschtes Antworten nicht ausgeschlossen werden können. Leider liegen hierzu keine Vergleichsinformationen aus dem Zeitraum vor Einführung des Entlassmanagements oder aus anderen Krankenhäusern vor. Somit lässt sich keine Aussage über eine Reduktion des Drehtüreffektes durch die strukturierte Überleitung bzw. ein Vergleich mit anderen Versorgern treffen.

## Schlussfolgerungen

Das Ziel aller am Überleitungsprozess beteiligten Parteien – Krankenhaus, Nachversorger, Hausarzt, Pflegedienst, Versicherer – muss der medizinisch-pflegerisch optimal versorgte, zufriedene Patient sein, der ohne Ängste die stationäre Behandlung verlässt. Darüber hinaus sind langfristig Einsparungen im Gesundheitssystem anzustreben, die durch ein optimales Netzwerk an zeitnahen Überleitungshilfen und Nachversorgern die Sicherheit und Gesundheit der Patienten gewährleisten. Die vorliegende explorative Studie liefert Ergebnisse zum Entlassmanagement in einer deutschen Universitätsklinik für Chirurgie, die unter anderem zeigen, dass die Verweildauer nach der gesetzlich vorgeschriebenen Einführung nicht gesunken ist.

Die Resultate dieser Analyse sind einerseits durch das spezielle, universitärchirurgische Kollektiv und andererseits durch die Kostenkalkulation basierend auf einer kleinen Subkohorte limitiert und selbstverständlich nur bedingt auf andere Kliniken oder Krankenhäuser übertragbar. Weitere prospektive Evaluationsstudien mit größeren Patientenkollektiven, insbesondere auch für andere klinische Fachbereiche sowie für die Kosten im nachstationären Sektor, sollten dringend durchgeführt werden, um im Rahmen von Verhandlungen zur Kostenvergütung als Bemessungsgrundlage zu dienen und die an der Überleitung beteiligten Partner optimal auszurichten.

Zur Sicherung der Strukturqualität wäre es hilfreich, für Krankenhäuser ausgewählte Assessmentinstrumente mit Mindestkriterien zur Erfassung der Risiken im Entlassmanagement festzulegen. Mit dieser Vorgabe wäre u. a. eine validere Messung als Grundlage für die Qualitätssicherung möglich.

## Supplementary Information





## Footnotes

1 Im Zeitraum vom 01.05.2016 bis 31.01.2017 wurde das Entlassmanagement stufenweise aufgebaut, daher fand in diesem Zeitraum keine vollumfängliche Versorgung statt und Patienten dieses Übergangszeitraumes wurden nicht einbezogen.
