# Supplementary material for: Implementation of discharge management in the surgical department of a university hospital: exploratory analysis of costs, length of stay, and patient satisfaction
Source: Bundesgesundheitsblatt Gesundheitsforschung Gesundheitsschutz. 2022 Feb 9;65(3):348–56. [Article in German] doi: 10.1007/s00103-022-03497-z (PMC8888510; doi:10.1007/s00103-022-03497-z)
Supplement: Supplementary file 1 [file 103_2022_3497_MOESM1_ESM.pdf]

Klinik und Poliklinik für Chirurgie
Klinikum rechts der Isar der Technischen Universität München
Anstalt des öffentlichen Rechts
Direktor: Univ.-Prof. Dr. med. H. Friess

# Analyse der Patientenzufriedenheit bezüglich des Entlassmanagements der Klinik und Poliklinik für Chirurgie zwei Jahre nach dessen Initiierung

Sophia Hassiotis, PD Dr. med. Volker Aßfalg, Prof. Dr. Jörg Königstorfer

**Hinweise zur Beantwortung der Fragen:**

Die Beantwortung der Fragen findet in Schulnoten von 1 – 6 statt. Wobei 1 für „sehr gut" bzw. „Stimme völlig zu" und 6 für „ungenügend" bzw. „Stimme überhaupt nicht zu" stehen.

---

**Fragebogeninterview:**

**Personenbezogenes:**

| Name, Vorname |
|---|
| (Etikett) |

1. Geschlecht:

☐ weiblich
☐ männlich

2. Alter:

_______ Jahre

3. Dauer des stationären Aufenthalts:

________ Tage
☐ kürzer als eine Woche
☐ ein bis zwei Wochen
☐ länger als zwei Wochen

4. Punktwert im BLAYLOCK-Assessment

__________ Punkte

5. Hauptdiagnose des stationären Aufenthaltes:

☐ Colon-/Rektumkarzinom
☐ Sigmadivertikulitis
☐ Magenkarzinom
☐ Ileus
☐ Ösophaguskarzinom
☐ HPB-Karzinom

6. Überleitungsindikation (Mehrfachnennung möglich):

☐ Stoma
☐ Wundheilungsstörung
☐ Grundpflege
☐ Ernährungstherapie
☐ Heimsauerstoff
☐ Behandlungspflege

7. Was hat der Patient erhalten:
(Mehrfachnennung mögl.)

☐ AHB
☐ Home Care Überleitung
☐ Pflegedienst
☐ Sonstiges:__________________________

**Evaluation Entlassmanagement**

1. Wussten Sie vor Ihrem Krankenhausaufenthalt, dass es im Krankenhaus ein sogenanntes Entlassmanagement gibt?

- ☐ Ja
- ☐ Nein

3. Wie gut wurden Sie von den Mitarbeiter/innen des Klinikums über die Aufgaben und Möglichkeiten des Entlassmanagements Ihrer Einschätzung nach informiert?

- ☐ 1 (sehr gut)
- ☐ 2 (gut)
- ☐ 3 (befriedigend)
- ☐ 4 (ausreichend)
- ☐ 5 (mangelhaft)
- ☐ 6 (ungenügend)

4. Wie empfanden Sie den Umgang des Personals des Entlassmanagements mit Ihnen, vom ersten Kontakt bis zur Entlassvisite?

- ☐ 1 (sehr gut)
- ☐ 2 (gut)
- ☐ 3 (befriedigend)
- ☐ 4 (ausreichend)
- ☐ 5 (mangelhaft)
- ☐ 6 (ungenügend)

5. Wie beurteilen Sie die Erreichbarkeit des Entlassmanagements?

- ☐ 1 (sehr gut)
- ☐ 2 (gut)
- ☐ 3 (befriedigend)
- ☐ 4 (ausreichend)
- ☐ 5 (mangelhaft)
- ☐ 6 (ungenügend)

6. Wie beurteilen Sie die Vorbereitung auf Ihre Entlassung aus dem Klinikum?

- ☐ 1 (sehr gut)
- ☐ 2 (gut)
- ☐ 3 (befriedigend)
- ☐ 4 (ausreichend)
- ☐ 5 (mangelhaft)
- ☐ 6 (ungenügend)

7. Wie gut hat die von den Seiten des Klinikums geplante Versorgung zu Hause dann wirklich funktioniert?

- ☐ 1 (sehr gut)
- ☐ 2 (gut)
- ☐ 3 (befriedigend)
- ☐ 4 (ausreichend)
- ☐ 5 (mangelhaft)
- ☐ 6 (ungenügend)

Inwiefern stimmen Sie den folgenden Aussagen zu? Bitte geben Sie den Grad Ihrer Zustimmung auf der Skala von 1 (stimme völlig zu) bis 6 (stimme überhaupt nicht zu).

8. Ich habe das Gefühl durch das Entlassmanagement eine hochqualitative Anschlussbehandlung erhalten zu haben.

- ☐ 1 (Stimme völlig zu)
- ☐ 2 (Stimme zu)
- ☐ 3 (Stimme eher zu)
- ☐ 4 (Stimme eher nicht zu)
- ☐ 5 (Stimme nicht zu)
- ☐ 6 (Stimme überhaupt nicht zu)

9. Ich habe das Gefühl durch das Entlassmanagement eine reibungslose Anschlussbehandlung erhalten zu haben.

☐ 1 (Stimme völlig zu)
☐ 2 (Stimme zu)
☐ 3 (Stimme eher zu)
☐ 4 (Stimme eher nicht zu)
☐ 5 (Stimme nicht zu)
☐ 6 (Stimme überhaupt nicht zu)

10. Ich bin ausreichend gut auf meine Entlassung vorbereitet worden.

☐ 1 (Stimme völlig zu)
☐ 2 (Stimme zu)
☐ 3 (Stimme eher zu)
☐ 4 (Stimme eher nicht zu)
☐ 5 (Stimme nicht zu)
☐ 6 (Stimme überhaupt nicht zu)

11. Haben Sie das Gefühl zum richtigen Zeitpunkt (weder zu früh noch zu spät) entlassen worden zu sein?

☐ 1 (Stimme völlig zu)
☐ 2 (Stimme zu)
☐ 3 (Stimme eher zu)
☐ 4 (Stimme eher nicht zu)
☐ 5 (Stimme nicht zu)
☐ 6 (Stimme überhaupt nicht zu)

12. Welche Schulnote von 1 bis 6 würden Sie dem Entlassmanagement insgesamt geben?

☐ 1 (sehr gut)
☐ 2 (gut)
☐ 3 (befriedigend)
☐ 4 (ausreichend)
☐ 5 (mangelhaft)
☐ 6 (ungenügend)

13. Waren Sie in den ersten 10 Tagen nach der Entlassung aus dem Klinikum bei Ihrem/Ihrer Hausarzt/-ärztin, niedergelassenen Facharzt/-ärztin oder in einer Krankenhausambulanz? (Mehrfachnennungen möglich)

☐ Hausarzt bzw. -ärztin/ Allgemeinarzt bzw. -ärztin
☐ Facharzt/-ärztin
☐ Krankenhausambulanz
    ☐ Klinikum rechts der Isar
    ☐ Anderes Klinikum: __________________

14. Waren Sie in den ersten 10 Tagen nach der Entlassung aus unserer Klinik **ungeplant** stationär in einem Krankenhaus?

☐ Ja
☐ Nein
    ☐ Klinikum rechts der Isar
    ☐ anderes Krankenhaus

wenn "ja“:

14a. Weswegen waren Sie stationär im Krankenhaus?

☐ Problem in Zusammenhang mit dem ersten Krankenhausaufenthalt
☐ anderes, unabhängiges Problem, nämlich: ____________________

15. Was hat Ihnen am besten am Entlassmanagement gefallen?

_______________________________________________________________________________

_______________________________________________________________________________

16. Was hat Ihnen am Entlassmanagement überhaupt nicht gefallen (z.B. was hat nicht funktioniert)?

_______________________________________________________________________________

_______________________________________________________________________________

17. Haben Sie Verbesserungsvorschläge für das Entlassmanagement?

_______________________________________________________________________________

_______________________________________________________________________________

Entlassdatum:

Datum der Datenerhebung:

Mitarbeiter/in:

Eingabe in die Datenbank:

Pseudonymisierung:

Mitarbeiter/in:
